# Language evaluation in children with pre-lingual hearing loss and cochlear implant^☆^^☆☆^

**DOI:** 10.1016/j.bjorl.2018.10.006

**Published:** 2018-11-22

**Authors:** Emille Mayara Scarabello, Dionísia Aparecida Cusin Lamônica, Marina Morettin-Zupelari, Liège Franzini Tanamati, Patrícia Dominguez Campos, Kátia de Freitas Alvarenga, Adriane Lima Mortari Moret

**Affiliations:** a Universidade de São Paulo (USP), Faculdade de Odontologia de Bauru (FOB), Departamento de Fonoaudiologia, Bauru, SP, Brazil; b Universidade de São Paulo (USP), Hospital de Reabilitação de Anomalias Craniofaciais (HRAC), Centro de Pesquisas Audiológicas (CPA), Seção de Implante Coclear, Bauru, SP, Brazil

**Keywords:** Hearing, Hearing loss, Cochlear implant, Child, Language, Audição, Perda auditiva, Implante coclear, Criança, Linguagem

## Abstract

**Introduction:**

The cochlear implant is an effective device for children with severe and/or profound prelingual hearing loss, since it provides considerable improvement in oral language acquisition through the auditory pathway. The use of a cochlear implant contributes to the development of auditory perception, favoring the acquisition of the linguistic processes related to communication skills, which might have a positive effect on other areas of development.

**Objective:**

The aim of this study was to verify the performance of children using cochlear implants for expressive and receptive oral language.

**Methods:**

This was a prospective cross-sectional study that used the following tests: Child language test in the phonology, vocabulary, fluency and pragmatics areas, and the Peabody picture vocabulary test. Thirty children participated in this study, of both genders, aged between 36 and 72 months, with severe and/or profound bilateral sensorineural hearing loss, without other impairments and users of unilateral cochlear implant with full electrode insertion for a minimum of 12 months.

**Results:**

The longer duration of the cochlear implant use, the younger age at surgery and the better performance in the auditory perception of speech influenced the performance in expressive and receptive oral language. Even though when compared to the normative language acquisition process, the results showed that these children had patterns of linguistic skills that are below their chronological age; the results indicate that these children are developing expressive and receptive oral language skills, and this is the outcome that should be taken into account in this study.

**Conclusion:**

The longer duration of the cochlear implant use, the younger age at surgery and the better performance in the auditory perception of speech influenced the performance in expressive and receptive oral language skills, but not in all the studied semantic categories.

## Resumo

**Introdução:**

O implante coclear é um tratamento eficaz para crianças com deficiência auditiva pré-lingual de grau severo e/ou profundo, uma vez que proporciona melhoria considerável na aquisição da linguagem oral por meio da via auditiva. O uso do implante coclear contribui para o desenvolvimento da percepção auditiva e favorece a aquisição dos processos linguísticos voltados à aquisição de habilidades comunicativas que poderá repercutir positivamente em outras áreas do desenvolvimento.

**Objetivo:**

Verificar o desempenho de crianças usuárias de implante coclear quanto à linguagem oral expressiva e receptiva.

**Método:**

Estudo prospectivo transversal onde os seguintes testes foram usados: teste de linguagem infantil nas áreas de fonologia, vocabulário, fluência e pragmática e *teste de vocabulário de imagens Peabody.* Participaram deste estudo 30 crianças de ambos os sexos de 36 a 72 meses, com deficiência auditiva sensorioneural de grau severo e/ou profundo bilateral, sem outros comprometimentos e usuárias de implante coclear unilateral com inserção total dos eletrodos havia, no mínimo, 12 meses.

**Resultados:**

O maior tempo de uso do implante coclear, a menor idade na cirurgia e o melhor desempenho na percepção auditiva da fala influenciaram o desempenho na linguagem oral expressiva e receptiva. Embora comparativamente ao processo de aquisição normativo da linguagem os resultados encontrados tenham revelado que essas crianças demonstraram habilidades linguísticas em padrões abaixo de sua idade cronológica, os resultados apontaram que essas crianças estão em evolução da linguagem oral expressiva e receptiva, esse é o resultado que deve ser valorizado neste estudo.

**Conclusão:**

O maior tempo de uso do implante coclear, a menor idade na cirurgia e o melhor desempenho na percepção auditiva da fala influenciaram o desempenho na linguagem oral expressiva e receptiva, porém não em todas as categorias semânticas estudadas.

## Introduction

The cochlear implant (CI) is an effective treatment device for children with severe and/or profound prelingual hearing loss, as it provides considerable improvement in the oral language acquisition through the auditory pathway.^1^ The differences reported in the studies reporting the results obtained for this population are extensive, and this may be related to the complex interaction of factors that start with the etiology of deafness, includes the cochlear implant indication criteria and involves the entire therapeutic process that aims for the development of auditory skills and oral language acquisition after the implantation.^2^

In Brazil, few studies of implanted children are related to oral language performance; overall, most of them report the results of the child's auditory performance. Basically, three reasons can be highlighted for the study of oral language outcomes in children after the cochlear implant: (1) Better delineation of the therapeutic process based on the child's performance; (2) Obtaining evolution parameters in groups of children using standardized tools, allowing the comparison of results; (3) Using the results as indicators of assessment of cochlear implant services.^3^, ^4^, ^5^

Considering all these facts, the aim of this study was to verify the performance of receptive and expressive oral language in children aged 3 years to 6 incomplete years with cochlear implants, correlated with audiological and demographic data.

## Methods

This is a prospective cross-sectional study performed at the Cochlear Implant Section of the Craniofacial Anomaly Rehabilitation Hospital of Universidade de São Paulo (IC-Sector-HRAC/USP), from July 2013 to March 2015, approved by the Research Ethics Committee (REC) of HRAC/USP CAAE n. 21571713.9.0000.5441. This study was financed in part by the Coordenação de Aperfeiçoamento de Pessoal de Nível Superior – Brasil (CAPES) – Finance Code 001.

Thirty children of both genders were included in this study, aged from 36 to 72 months, with severe and/or profound bilateral sensorineural hearing loss, without other impairments and who had been users of unilateral CI with full electrode insertion for a minimum of 12 months.

Demographic data included gender, age at evaluation, age at surgery and hearing age (time of cochlear implant use). The results of the procedures performed during the child's last follow-up in the service were analyzed.

The audiological data and the studied and correlated data of speech perception were collected by analyzing the charts of each participating child. The minimum response level of open-field audiometry, the phoneme recognition rate,^6^ the distribution of participants according to the Hearing Categories^7^ and the Infant-Toddler Meaningful Auditory Integration Scale (IT-MAIS),^8^, ^9^ which consists of a structured interview to evaluate the child's spontaneous responses to the sounds of their daily environment, were considered. The evaluation is based on the information provided by the child's parents and/or caregivers in 10 simple questions that evaluate three main areas as follows: the behavior of vocalization, the attention responses to environmental and speech sounds and sound recognition. All the questions had five alternative answers, to which the evaluator assigns points according to the examples provided by the parents and/or caregivers, generating, in the end a scaled result in percentages.

For the evaluation of expressive oral language, the ABFW Child Language Test-Part B – Vocabulary^10^ was used, which qualitatively and quantitatively assesses the expressive vocabulary in nine conceptual fields: clothing, animals, foods, means of transportation, furniture and utensils, professions, places, shapes and colors, toys and musical instruments.

For the evaluation of the receptive oral language, the Peabody picture vocabulary test Hispanic-American adaptation (TVIP) was used,^11^ which evaluates the lexical development in the receptive domain, providing objective and accurate information about the receptive-auditory vocabulary in a wide variety of areas (personal, actions, qualities, body parts, time, nature, places, objects, animals, tools and instruments).

To obtain the correlation between demographic variables, audiological data and speech perception with the applied tools, the following statistical tests were used: Kolmogorov–Smirnov Normality Test, Pearson's Correlation Test and Spearman's Correlation Test.

## Results

Table 1 shows the demographic data on gender, age at evaluation, age at surgery and time of cochlear implant use. Table 2 shows the minimum level of open-field audiometry response, the phoneme recognition rate,^6^ the Infant-Toddler Meaningful Auditory Integration Scale (IT-MAIS),^7^, ^8^ and the participants’ distribution according to the Hearing Categories.^9^ We considered the results of the tests performed during the child's last follow-up visit at the service.

**Table 1:** Demographic data of 30 children related to gender, age at evaluation, age at surgery and time of cochlear implant use.

	*N*	%
Gender		
Female	20	(66.6)
Male	10	(33.4)

Age at evaluation		
Months		
36–48	7	(23.3)
49–60	13	(43.4)
61 to <72	10	(33.4)

Age at surgery		
Months		
≤12	1	(3.4)
13–24	12	(40.0)
25–36	17	(56.6)

Time of cochlear implant use		
Months		
12–24	9	(30.0)
25–36	8	(26.6)
37–48	10	(33.4)
49–60	3	(10.0)

**Table 2:** Minimum level of response at the open-field audiometry, phoneme recognition rate, Infant-Toddler Meaningful Auditory Integration Scale (IT-MAIS) and hearing categories of the 30 studied children.

	Median	Minimum	Maximum	SD
Audiometry (dB)	35.5	25.0	45.0	5.5
Phoneme recognition rate (%)	76.3	46.25	95.0	11.7
IT-MAIS	86.9	13.0	100.0	23.5

Hearing categories	0	1	2	3	4	5	6	Total
	–	2	4	2	12	10	–	30
	–	6.7%	13.3%	6.7%	40%	33.3%	–	100%

Table 3 shows the correlation between demographic variables and speech perception data in relation to the data obtained in the ABFW Child Language Test-Part B – Vocabulary. A statistically significant difference was observed for the UVD (Usual Verbal Designations) mechanism when comparing the duration of device use and the categories “Animals”; “Foods”, “Means of Transportation” and “Professions”, The longer the duration of device use, the better the results of expressive oral language in these categories.

**Table 3:** Correlation values between the demographic variables and speech perception data in relation to the data obtained from the ABFW Child Language Test^10^ – for the Usual Verbal Designation (UVD), No Designation (ND) and Substitution Process (SP) mechanisms.

		Age at surgery (*p*)	Age at evaluation *r* (*p*)	Time of implant use *r* (*p*)	Phonemes (%) *r* (*p*)	Words (%) *r* (*p*)	IT-MAIS *r* (*p*)	Hearing category *r* (*p*)
UVD	Clothing	−0.302 (0.184)	0.252 (0.270)	0.409 (0.065)	0.624 (0.003^a^)	0.619 (0.004^a^)	0.270 (0.237)	0.639 (0.002^a^)
	Animals	−0.424 (0.044^a^)	0.163 (0.458)	0.421 (0.046^a^)	0.327 (0.138)	0.196 (0.383)	0.326 (0.129)	0.519 (0.011^a^)
	Foods	−0.413 (0.050^a^)	0.276 (0.202)	0.515 (0.012^a^)	0.466 (0.029^a^)	0.527 (0.012^a^)	0.357 (0.094)	0.745 (0.000^a^)
	Means of transportation	−0.330 (0.134)	0.276 (0.215)	0.455 (0.033^a^)	0.370 (0.098)	0.399 (0.073)	0.344 (0.117)	0.508 (0.016^a^)
	Furniture and utensils	−0.285 (0.198)	0.166 (0.460)	0.329 (0.135)	0.649 (0.001^a^)	0.679 (0.001^a^)	0.326 (0.138)	0.662 (0.001^a^)
	Professions	−0.192 (0.416)	0.457 (0.043^a^)	0.462 (0.040^a^)	0.411 (0.081)	0.398 (0.092)	0.228 (0.334)	0.347 (0.133)
	Places	−0.108 (0.652)	0.346 (0.135)	0.330 (0.155)	0.704 (0.001^a^)	0.742 (0.000^a^)	0.462 (0.040^a^)	0.759 (0.000^a^)
	Shapes and colors	−0.286 (0.209)	0.218 (0.343)	0.377 (0.092)	0.707 (0.000^a^)	0.635 (0.003^a^)	0.464 (0.034^a^)	0.827 (0.000^a^)
	Toys and musical instruments	−0.294 (0.196)	0.090 (0.698)	0.259 (0.257)	0.533 (0.016^a^)	0.586 (0.007^a^)	0.214 (0.352)	0.445 (0.043^a^)
ND	Clothing	0.529 (0.014^a^)	0.135 (0.559)	−0.259 (0.256)	−0.360 (0.118)	−0.283 (0.227)	0.046 (0.844)	−0.053 (0.821)
	Animals	0.229 (0.293)	−0.189 (0.387)	−0.337 (0.116)	−0.356 (0.104)	−0.325 (0.140)	−0.677 (0.000^a^)	−0.651 (0.001^a^)
	Foods	0.366 (0.086)	−0.169 (0.441)	−0.417 (0.048^a^)	−0.566 (0.006^a^)	−0.495 (0.019^a^)	−0.493 (0.017^a^)	−0.339 (0.113)
	Means of transportation	0.190 (0.398)	−0.140 (0.534)	−0.270 (0.223)	−0.447 (0.042^a^)	−0.474 (0.030^a^)	−0.366 (0.094)	−0.352 (0.108)
	Furniture and utensils	0.463 (0.030^a^)	−0.093 (0.682)	−0.424 (0.049^a^)	−0.547 (0.010^a^)	−0.497 (0.022^a^)	−0.464 (0.029^a^)	−0.410 (0.058^a^)
	Professions	0.526 (0.017^a^)	−0.128 (0.590)	−0.488 (0.029^a^)	−0.501 (0.029^a^)	−0.347 (0.146)	−0.602 (0.005^a^)	−0.303 (0.194)
	Places	0.313 (0.179)	−0.511 (0.021^a^)	−0.628 (0.003^a^)	−0.755 (0.000^a^)	−0.749 (0.000^a^)	−0.526 (0.017^a^)	−0.643 (0.002^a^)
	Shapes and colors	0.320 (0.157)	−0.326 (0.149)	−0.493 (0.023^a^)	−0.721 (0.000^a^)	−0.615 (0.004^a^)	−0.490 (0.024^a^)	−0.805 (0.000^a^)
	Toys and musical instruments	0.412 (0.063)	0.024 (0.917)	−0.270 (0.237)	−0.459 (0.042^a^)	−0.419 (0.066)	−0.364 (0.105)	−0.142 (0.539)
SP	Clothing	−0.175 (0.448)	−0.376 (0.093)	−0.178 (0.439)	−0.311 (0.183)	−0.366 (0.112)	−0.298 (0.189)	−0.546 (0.010^a^)
	Animals	0.383 (0.071)	−0.115 (0.601)	−0.336 (0.117)	−0.142 (0.527)	−0.020 (0.928)	−0.002 (0.994)	−0.235 (0.281)
	Foods	0.353 (0.108)	−0.013 (0.953)	−0.246 (0.269)	−0.029 (0.902)	0.039 (0.867)	−0.124 (0.583)	−0.396 (0.068)
	Means of transportation	0.353 (0.108)	−0.013 (0.953)	−0.246 (0.269)	−0.029 (0.902)	0.039 (0.867)	−0.124 (0.583)	−0.396 (0.068)
	Furniture and utensils	−0.020 (0.928)	−0.117 (0.604)	−0.057 (0.802)	−0.313 (0.168)	−0.383 (0.087)	−0.024 (0.914)	−0.440 (0.040^a^)
	Professions	−0.448 (0.048^a^)	−0.108 (0.652)	0.258 (0.272)	0.330 (0.168)	0.067 (0.786)	0.592 (0.006^a^)	0.118 (0.620)
	Places	−0.271 (0.248)	0.517 (0.019^a^)	0.612 (0.004^a^)	0.669 (0.002^a^)	0.581 (0.009^a^)	0.560 (0.010^a^)	0.567 (0.009^a^)
	Shapes and colors	−0.180 (0.435)	0.354 (0.115)	0.423 (0.056^a^)	0.204 (0.388)	0.105 (0.659)	0.217 (0.344)	0.205 (0.373)
	Toys and musical instruments	−0.044 (0.850)	−0.097 (0.677)	−0.028 (0.905)	−0.152 (0.521)	−0.242 (0.304)	0.081 (0.726)	−0.315 (0.164)

*r*, correlation coefficient; *p* (*p* value), statistically significant result.

a Statistically significant difference, considering *p* < 0.05.

Regarding the age at surgery, a statistically significant difference was observed for the categories “Animals” and “Foods”, but with a negative correlation; that is, the younger the age at surgery, the higher the scores in these categories.

Also in Table 3, the correlation of the UVD mechanism with the speech perception data (Speech and Phoneme recognition rate, Infant-Toddler Meaningful Auditory Integration Scale IT-MAIS and Hearing Categories) showed a statistically significant difference with a positive correlation in the categories “Clothing”, “Foods”, “Furniture” and “Utensils”, “Shapes and Colors”, “Toys and Musical Instruments”, especially when correlated with the Hearing Categories, showing that the better the speech perception performance, the better the results of expressive vocabulary.

As for the Non-Designation (ND) mechanism (the score occurs when the participant does not name the target word), the results showed a statistically significant difference for the semantic categories “Clothing”, “Furniture and Utensils” and “Professions” in relation to the age at surgery, showing that the older the age at surgery, the higher the score in the Non-Designation mechanism.

There was also a statistically significant difference for some semantic categories when correlated to the time of device use, with a negative correlation, i.e., the shorter the time of cochlear implant use, the higher the Non-Designation score (Table 3).

For the correlation of this same mechanism (Non-Designation – ND) and speech perception data (Speech and Phoneme recognition rate, Infant-Toddler Meaningful Auditory Integration Scale – IT-MAIS and Hearing Categories), it was found that for the “Animals”, “Foods”, “Means of Transportation”, “Furniture and Utensils”, “Professions” “Places”, “Shapes and Colors”, “Toys and Musical Instruments”, there was a statistically significant difference with a negative correlation; that is, the worse the performance in speech perception, the higher the scores for the Non-Designation (ND) mechanism (Table 3).

In the correlation between the age at the evaluation, time of cochlear implant use and the perception of speech with the semantic categories in the Substitution Process (SP) (the mechanism that the child uses to replace the target word), there was a statistically significant difference in several semantic categories, with a positive correlation.

Table 4 shows the distribution of the participating children, in percentages, in relation to the attained classification (non-testable, above, equal to or below the expected for normality) in the Usual Verbal Designations mechanism.

**Table 4:** Distribution of children, in percentage, in relation to the classification obtained (non-testable, above, equal to or below the expected for normality) in the Verbal Designations – ABFW (Part B) mechanism.^10^

Usual Verbal Designation										
	Clothing	Animals	Foods	Means of transportation	Furniture and utensils	Professions	Places	Shapes and colors	Toys and musical instruments	Value obtained in
Below the expected	56.6	33.3	56.6	53.3	66.6	56.6	66.6	36.6	63.3	%
Equal to the expected	0	6.6	3.3	0	0	3.3	0	3.4	0	%
Above the expected	10	36.6	16.6	20	6.6	6.6	0	30	6.7	%
Non-testable^a^	33.4	23.4	23.4	26.7	26.7	33.4	33.4	30	30	%

a The child did not perform the requested task because they did not understand the test.

Table 5 shows the quantitative values of mean, minimum, maximum and standard deviation, in categories and correlation values between demographic variables and speech perception data in relation to data obtained from the Peabody picture vocabulary test: Hispanic-American adaptation,^11^ which showed a statistically significant difference with a negative correlation only for age at evaluation; that is, younger children performed better in this test.

**Table 5:** Quantitative mean, minimum, maximum and standard deviation values in categories and correlation values between demographic variables, audiological data and speech perception data in relation to data obtained with the Peabody picture vocabulary test: Hispanic-American adaptation.^11^

	Mean	Minimum	Maximum	Standard deviation	Age at surgery *r* (*p*)	Age at evaluation *r* (*p*)	Time of implant use *r* (*p*)	Threshold pure-tone audiometry *r* (*p*)	Phonemes (%) *r* (*p*)	Words (%) *r* (*p*)	IT-MAIS *r* (*p*)	Hearing category *r* (*p*)
Peabody	1.83	0.00	4.00	0.93	−0.297 (0.168)	−0.645 (0.001^a^)	−0.332 (0.122)	0.194 (0.374)	0.020 (0.929)	0.012 (0.957)	−0.252 (0.246)	0.047 (0.831)

*r*, correlation coefficient; *p* (*p* value), statistically significant result.

a Statistically significant difference, considering *p* < 0.05.

## Discussion

All children in this study had the minimum required audibility, ensuring access to the auditory perception of speech, represented by the values threshold audiometry in the open-field test with cochlear implants, phoneme and word recognition rate, performance in the Infant-Toddler Meaningful Auditory Integration Scale – IT-MAIS and in the auditory categories (Table 2). However, what can be observed is a variability of oral language results obtained by the children. The literature also describes great variability of results in expressive language skills.

Initially, regarding the age at surgery, it was observed that the younger the age at the time of surgery, the more satisfactory the results for oral language (Table 3). The literature also addresses this aspect.^3^, ^12^, ^13^, ^14^, ^15^, ^16^, ^17^, ^18^, ^19^, ^20^ However, the impact of hearing impairment results in a delay in oral language acquisition even in children implanted during the sensitive period of neuronal plasticity. In this study, more than half (50%) of the children showed a performance that was below the expected outcome when compared to the normative test (hearing pairs) for age in seven of the nine evaluated semantic categories, as observed in Table 4, which shows the performance in expressive skills of the Usual Verbal Designation mechanism.

Variability of results was observed regarding the time of device use, that is, the longer the time of cochlear implant use, the better the results in expressive oral language in the semantic categories “Animals”; “Foods”, “Means of Transportation” and “Professions” in the UVD mechanism (Table 3). The significant correlation between the auditory perception of speech and the expressive vocabulary was expected, given that the studies in the literature broadly indicate how much the auditory perception of speech contributes to advances in expressive language skills. This correlation was found in eight of the nine studied semantics categories. The children who were older at surgery did not perform well in naming words of the semantic categories “Clothing”, “Furniture and Utensils” and “Professions” (ND mechanism). Although this result corroborates the literature, which indicates that the older the age at surgery, the worse the results in oral language performance,^13^, ^16^ this is a careful analysis. It is known that the semantic acquisition of these categories generally occurs at older ages during the language acquisition process in children with typical development.^21^ All the children evaluated in this study were between the ages of 3 and 6 years, that is, still in the period of oral language development. The fact that they did not have the verbal designation capacity for words of semantic categories that are acquired later might not mean poor performance, but that this ability is still occurring, considering that the implanted children have a late hearing age. The same reasoning applies to the statistically significant difference for some semantic categories when they are correlated to the time of device use, with a negative correlation; that is, the shorter the time of cochlear implant use, the higher the Non-Designation (ND) score. The time of device use represents the child's hearing age, meaning that children with less time of cochlear implant use are those with the lower hearing age and shorter time of exposure to the oral language through the auditory perception of speech.^12^

Another relevant aspect regarding the time of cochlear implant use and the Non-Designation (ND) should be pointed out. Non-designation (ND) is considered to be a worse performance in expressive oral language. The fact that this occurred with children with shorter time of CI use might mean that these children have not yet achieved enough minimum time of auditory access to speech sounds to show qualitative leaps in oral language performance. Therefore, it does not necessarily mean an unsatisfactory result. It should be remembered that implanted children with prelingual hearing loss have auditory access to speech perception at a later age, and not at birth, as it occurs with children with normal hearing. This fact alone results in a dislocation of the beginning of the oral language acquisition to older ages, and the entire process of oral language acquisition and development may be delayed. The studied children may have still incipient results in expressive oral language, but it does not mean that the acquisition is not occurring. The study by Percy-Smith et al.^14^ also reports on this subject.

Children need the receptive process before being able to express themselves, that is, it is predicted, in the typical language development, that comprehension skills precede expressive ones.^22^ These findings can be confirmed by the statistically significant difference with a negative correlation of this same mechanism (Non-Designation – ND) with the speech perception data (Phoneme and Speech Recognition Rate; the Infant-Toddler Meaningful Auditory Integration Scale – IT-MAIS and Hearing categories) in eight semantic categories. The worse the performance in speech perception, the higher the Non-Designation (ND) scores (Table 3). However, it should be recalled that oral language acquisition and development in children with prelingual hearing loss are highly vulnerable to many factors, and not exclusively to the access to auditory perception of speech sounds.

As for the Substitution Process (SP), there was a statistically significant difference in several semantic categories (Table 3), with a positive correlation, indicating that the older the participants, the longer the duration of the cochlear implant use and the higher the speech perception score, the greater the occurrence of the substitution process, which is qualitatively better than the Non-Designation (ND). The use of substitutions instead of Usual Verbal Designation (UVD) obviously means expressive oral language delay; however, the presence of this process represents an advance in communication skills, as the provided answers had a semantic association with the expected target word, for instance, “ship” in place of “boat”. This could facilitate their communication interaction with the speaker in conversation situations.^23^

All children in this study were implanted in the pre-lingual phase and, therefore, were vulnerable to the impact of hearing impairment on oral language and the several aspects that interfere with the cochlear implant outcome. Thus, children who were implanted at younger ages, younger children at the evaluation and those with a shorter time of cochlear implant use and still incipient speech perception performance, had an evolving vocabulary and clearly showed linguistic performance to fill the gap of the verbal concept of something they understand, but still do not know how to name. The fact that these children show this linguistic effort is a positive outcome and may favorably reflect on the acquisition of verbal concepts and the usual verbal designation in the future.

Regarding the receptive skills, the younger children at the evaluation showed a better statistically significant performance in the Peabody picture vocabulary test: Hispanic-American adaptation (TVIP) (Table 5). Colalto et al. also found similar results in their study.^24^

Although the age at surgery was not statistically significant, the correlation with the results of this tool was a negative one (Table 5), which means that the younger the age at surgery, the better the performance in these skills.^25^ The cochlear implant literature confirms that the best performance in speech perception and oral language is related to the performance of the surgery at early ages.^12^, ^14^, ^15^, ^16^, ^17^, ^19^, ^20^, ^26^ Thus, it is worth mentioning the degree of difficulty imposed by the Peabody picture vocabulary test: Hispanic-American adaptation (TVIP). In this tool, there is a demand for knowledge of the verbal concept in one-dimensional material (pictures presented during the test application) and not objects or concrete items, choosing one among four stimuli. Another noteworthy aspect refers to the need for linguistic knowledge of the pictures that represent an action. In this tool, the verbs are presented in the infinitive form. As an example: for the picture of a girl reading a book, the word presented is “read”. It is natural for children to have more difficulty with infinitive verbs, since in everyday life, considering the Portuguese language spoken in Brazil, the occurrence of conjugated verbs is greater.

The analysis of the minimum level of response in the open-field test with cochlear implants in Threshold Pure-Tone Audiometry, Word and Phoneme Recognition Rate and the Infant-Toddler Meaningful Auditory Integration Scale (IT-MAIS), showed that the higher the performance in speech perception, the better the performance in oral language. Regardless of the used tool, studies also indicate this same association between performance in speech perception and oral language in implanted children.^27^, ^28^

Although the obtained results showed that the assessed children had linguistic skills that were below their chronological age in comparison with the normative process of oral language acquisition, these results provide evidence that these children are still developing their receptive and expressive oral language.

## Conclusions

The longer duration of cochlear implant use, the younger age at surgery and the better performance at speech auditory perception influenced the receptive and expressive oral language performance in most of the studied semantic categories.

## Funding

Coordenação de Aperfeiçoamento de Pessoal de Nível Superior (CAPES).

## Conflicts of interest

The authors declare no conflicts of interest.
